# Developing a web-based SKOS editor

**DOI:** 10.1186/s13326-015-0043-z

**Published:** 2016-04-04

**Authors:** Mike Conway, Artem Khojoyan, Fariba Fana, William Scuba, Melissa Castine, Danielle Mowery, Wendy Chapman, Simon Jupp

**Affiliations:** Department of Biomedical Informatics, University of Utah, 421 Wakara Way, Salt Lake City, 84108 UT United States; Independent software developer, Kyiv, Ukraine; CALIT2, University of California San Diego, 9500 Gilman Drive, La Jolla, 92093 CA United States; European Bioinformatics Institute, Hinxton, CB10 1SD Cambridgeshire United Kingdom

## Abstract

**Background:**

The Simple Knowledge Organization System (SKOS) was introduced to the wider research community by a 2005 World Wide Web Consortium (W3C) working draft, and further developed and refined in a 2009 W3C recommendation. Since then, SKOS has become the *de facto* standard for representing and sharing thesauri, lexicons, vocabularies, taxonomies, and classification schemes. In this paper, we describe the development of a web-based, free, open-source SKOS editor built for the development, curation, and management of small to medium-sized lexicons for health-related Natural Language Processing (NLP).

**Results:**

The web-based SKOS editor allows users to create, curate, version, manage, and visualise SKOS resources. We tested the system against five widely-used, publicly-available SKOS vocabularies of various sizes and found that the editor is suitable for the development and management of small to medium-size lexicons. Qualitative testing has focussed on using the editor to develop lexical resources to drive NLP applications in two domains. First, developing a lexicon to support an Electronic Health Record-based NLP system for the automatic identification of pneumonia symptoms. Second, creating a taxonomy of lexical cues associated with Diagnostic and Statistical Manual of Mental Disorders (DSM-5) diagnoses with the goal of facilitating the automatic identification of symptoms associated with depression from short, informal texts.

**Conclusions:**

The SKOS editor we have developed is — to the best of our knowledge — the first free, open-source, web-based, SKOS editor capable of creating, curating, versioning, managing, and visualising SKOS lexicons.

**Electronic supplementary material:**

The online version of this article (doi:10.1186/s13326-015-0043-z) contains supplementary material, which is available to authorized users.

## Background

The Simple Knowledge Organization System (SKOS) standard was introduced to the wider community by a 2005 World Wide Web Consortium (W3C) working draft [1], and further developed and refined in a 2009 W3C recommendation [2, 3]^1^. Since then, SKOS has become the *de facto* standard for representing thesauri, lexicons, vocabularies, taxonomies, and classification schemes, both as a useful data format in its own right, and as a means for sharing resources on the semantic web. In this paper, we describe the development of a web-based, free, open-source SKOS editor suitable for the creation and curation of knowledge organization systems in general, and health-related lexicons designed to support clinical Natural Language Processing (NLP) in particular.

SKOS is a flexible standard designed to represent and encode a wide number of different types of knowledge organization systems, including vocabularies, thesauri, and classification systems. The standard is widely used by governments [4] (e.g. *United Kingdom Public Sector Vocabularies, French National Library Subject Headings, United States Library of Congress Subject Headings*), scientific bodies (e.g. *International Virtual Observatory Alliance Astronomy Vocabulary*, *NASA vocabularies*, *Thesaurus for the Social Sciences*), and non-governmental organisations (e.g. *Wikipedia categories*, *UNESCO Thesaurus*, *General Multilingual Environmental Thesaurus*). In contrast to its sibling World Wide Web Consortium semantic web standard, the Web Ontology Language (OWL), SKOS follows the principle of “minimal ontological commitment” [3]. That is, SKOS concepts and relations are lightly specified, using thesaurus-style relations like “broader” rather than logically formalised relations commonly used in OWL (e.g. *IS_A*).

SKOS models consist of *concept schemes* which serve as containers for *concepts*. Concepts can be related together in various ways to create a hierarchical structure. The most important of these semantic relations are:

- skos:broader can be read as “has broader concept”. For instance, the relation Photophobiaskos:broaderVisionProblem, asserts that Photophobia has broader concept VisionProblem.
- skos:narrower which can be read as “has narrower concept”. For instance, the relation VisionProblem
  skos:narrowerPhotophobia, asserts that VisionProblem has narrower concept Photophobia.
- skos:related can be read as “is related to”. For instance, the relation Photophobiaskos:related
  Diplopia, asserts that Photophobia is related to Diplopia.

Each SKOS concept can be associated with several types of lexical labels:

- skos:prefLabel (*preferred label*) provides a mechanism to link a preferred label to a concept. The prefLabel is the primary means of referring to a concept. Only one prefLabel per language should be assigned to each concept. For example, the SKOS concept Fever could have the skos:prefLabel“fever”@en (note that “@en” refers to English language).
- skos:altLabel (*alternative label*) provides a mechanism to specify synonyms or near-synonyms for a given concept. For example, the concept Fever could have the skos:altLabel“febrile”@en. This relation is especially useful for specifying synonymous terms necessary for NLP.
- skos:hiddenLabel (*hidden label*) provides a mechanism to specify non-standard synonymous terms (e.g. misspellings, typographical errors). For example, the concept Fever could have the skos:hiddenLabel“feber”@en. Hidden labels are particularly useful for encoding common misspellings necessary for NLP systems.

In addition to the semantic relations and lexical labels described above, SKOS also provides facilities to add additional metadata to concepts and map SKOS concepts to external vocabularies.

Given its lightweight semantics, SKOS is particularly suitable as a basis for the development and sharing of vocabularies to support NLP tasks. A key part of the workflow in developing some NLP systems – in particular NLP systems designed to process health-related text – is the development of custom lexicons, including common abbreviations, synonyms (including slang terms), and truncations [5–8].

Since its inception in 2005, significant effort has been expended on the development of software tools for the SKOS standard, in particular in editing and viewing SKOS vocabularies. Whilst OWL editors, such as Protégé^2^, can be used to create and edit SKOS, they require a user to understand SKOS in terms of OWL; an unnecessary overhead for a user simply interested in creating SKOS. Furthermore, a major requirement for a SKOS editing tool is the ability to visualise and navigate SKOS concept scheme broader/narrower hierarchies, a functionality that is unlikely be supported by generic OWL and RDF (Resource Description Framework) tools. Notable examples of “SKOS aware” tools include a SKOS Application Programming Interface (API) and editing module [9] for Protégé 4^3^ (the Protégé SKOS Editor), PoolParty, an online SKOS editing and manipulation tool [10], and SKOS functionality built into the TopBraid Composer RDF editing platform [11]^4^, all of which facilitate the creation, development, and utilisation of SKOS vocabularies. However, to the best of our knowledge, until now no free, open-source, web-based SKOS editor has been available to the research community (note that PoolParty, although web-based, is a commercial product). In this paper, we present a web-based SKOS editing tool that is suitable for developing and modifying the health-related lexicons necessary for large-scale information extraction from clinical notes and other health-related text, yet is also general purpose enough for any small-to-medium-sized SKOS vocabulary development or curation project.

## Implementation

A key advantage of using a web-based editor, is that it can be used anywhere, on any machine, without complex user installation. Given that our target users are clinicians, public health workers, and domain experts — i.e. those with little or no experience of semantic web languages — rather than informatics professionals, ease of use is an important requirement. We took the decision to simplify the editor’s user interface as much as possible, hiding some of the general OWL/RDF functionality available in tools like Protégé and TopBraid Composer.

**Fig. 1:**
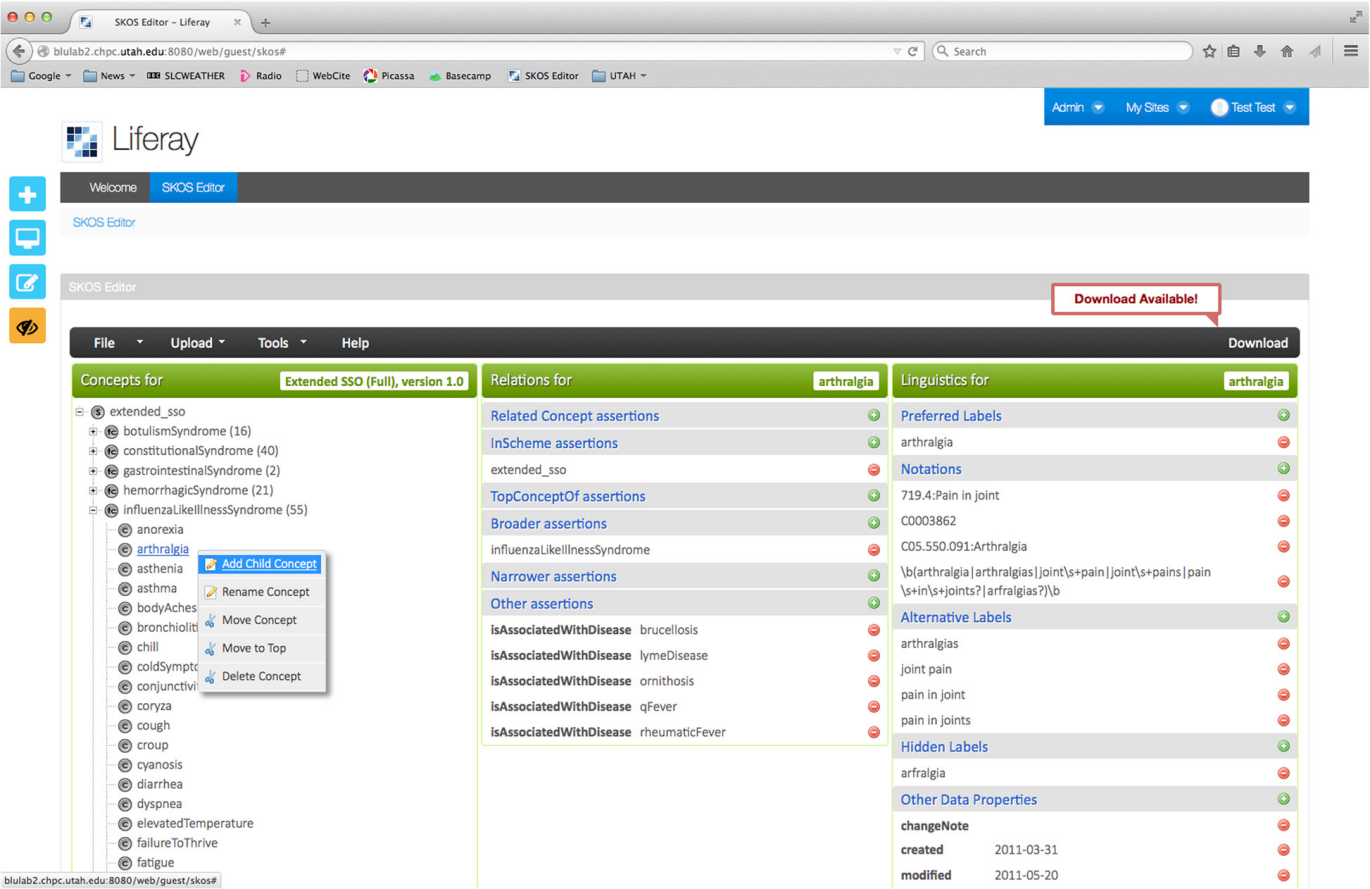
Screenshot of the system interface showing a biosurveillance lexical resource, with a “new concept” pop-up
- Relations Pane: An editable list of relations between concepts, particularly the skos:related, skos:broader, and skos:narrower relations
- Linguistics Pane: An editable list of lexical items related to each SKOS concept (e.g. skos:prefLabel, skos:altLabel, skos:hiddenLabel)

Considerable effort was expended on designing the user interface (a screenshot of the system is shown in Fig. 1 showing a SKOS thesaurus designed to drive a NLP system for the automatic identification of biosurveillance-relevant symptoms from Electronic Health Records (EHRs) [12]). After some experimentation, we adopted an interface that consists of three panes, from left to right:

- Concept Pane: An editable taxonomic hierarchy of SKOS concepts representing skos:broader and skos:narrower relations, which the user can click on to expand and collapse the tree

We identified six core functionalities necessary for the editor, partially based on the requirements identified by [9]:

- Create, edit, and delete SKOS entities
- Assert SKOS relationships between SKOS concepts (e.g. broader/narrower)
- Assert and edit skos:prefLabel, skos:altLabel, and skos:hiddenLabel data properties
- Visualise broader and narrower relationships in a browsable hierarchical tree
- Support for SKOS documentation properties
- Provide alternative renderings (e.g. multilingual prefLabels) within the editor

Additionally, our editor provides versioning, and a Wizard tool to expedite the SKOS concept hierarchy creation process.

In building our web-based SKOS Editor, we relied heavily on existing OWL, SKOS and RDF tooling, in particular, the SKOS API [9] (developed by author Jupp) and the OWL API [13]. The system is a Liferay portlet application that uses a standard **M**odel-**V**iew-**C**ontroller architecture implemented using the following technologies:

- Business (Model) Layer: Java SKOS API and OWL API
- Presentation (View) Layer: JavaScript/JSP/JQuery Libraries provides a rich web 2.0 user interface connected to the middle layer via AJAX calls
- Controller/Middle Layer: The Liferay Portlet application using the JSR 286 Portlet framework connects the presentation layer to the SKOS API, as well as providing user management, authorisation, and authentication.

A MySQL database is used to save files and file versions, as well as user specific settings. The application is a Single Page Application, with all server/client communication based on Ajax calls using a JQuery library (client-side) and Liferay portlet (server-side).

**Fig. 2:**
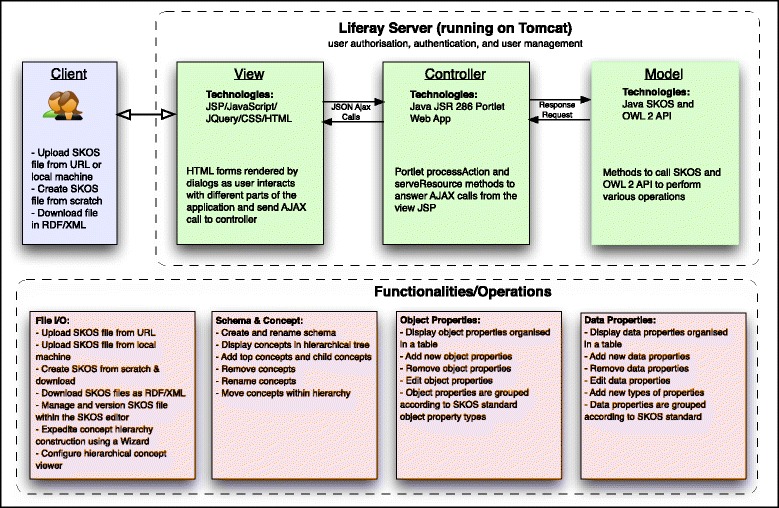
Flowchart describing system functionality

A screenshot of the system interface is shown in Fig. 1 and a diagram representing the system architecture is shown in Fig. 2.

## Results and discussion

**Fig. 3:**
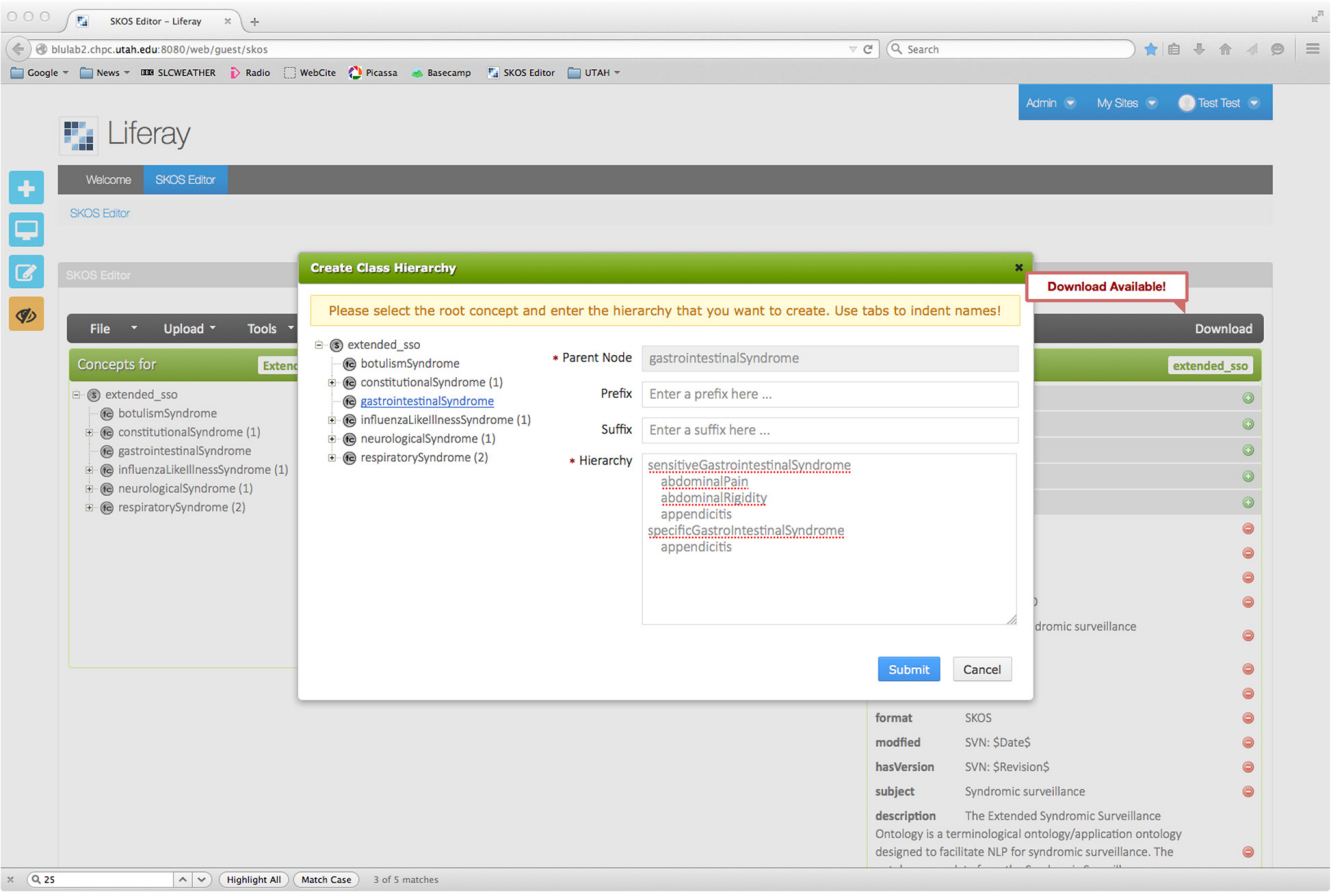
Concept creation Wizard designed to expedite the creation of SKOS concept hierarchies

The web-based SKOS editor allows a user to upload a SKOS file from their local machine for editing, load a SKOS file from a URL, create a SKOS file *ab initio*, and download an edited SKOS file to a local machine. Furthermore, the editor supports versioning of SKOS files, and provides a GUI-based “Wizard” to expedite the creation of concept hierarchies. The Wizard allows a user to input a plain-text tab indented concept hierarchy, a functionality that has been shown in our qualitative user testing to expedite the hierarchy creation process (see Fig. 3 and Additional file 1). The tool takes its inspiration from the Protégé SKOS editor developed by author Jupp, and supports core SKOS functionalities. In the “Concept Pane”, SKOS concept schemes and concepts can be created and manipulated with a hierarchical tree structure. The “Relation Pane” shows hierarchical relations defined in the concept pane, and allows these relations to be modified, including the addition of non-hierarchical relations between concepts. The “Linguistics Pane” allows lexical information — prefLabels, hiddenLabels, altLabels — to be associated with each concept.

While there have been attempts at developing best practices for SKOS thesauri development (e.g. [3, 14]) considerable heterogeneity exists between different SKOS resources [15]. We built an editor that is designed to handle even those SKOS resources that do not adhere to suggested best practice (e.g. the thesauri has more than one prefLabel for a specified language, or a SKOS concept exists outside a Concept Scheme).

### Loading and editing sample SKOS vocabularies

In order to demonstrate and test the capacities of the SKOS editor, we tested the performance of the editor in executing some key editing functions. To test the editor, we used an Apple MacBook with 16GB of memory and the Firefox web browser (version 32). We chose five widely used SKOS resources:

- STW for Economics Thesaurus is used for indexing economics research papers [16]
- New York Times (NYT) Subject Descriptions is used to index NYT news stories [17]
- United Kingdom Archive Thesaurus is a general purpose subject heading thesaurus developed by the UK government [18]
- Australian Curriculum Thesaurus, a resource developed by the Australian government for managing educational resources [19]
- The UNESCO — United Nations Educational, Scientific and Cultural Organization — Thesaurus provides general subject terms across the fields of education, culture, natural science, social and human sciences, communication, and information [20]

Table 1 shows the capabilities of the editor in editing large thesauri, where it can be seen that the 5.1 MB *UNESCO Thesaurus* took six seconds to load into the tool. However, larger thesauri – e.g. the *UK Archive Thesaurus* at 9.4 MB – do not load quickly due to limitations within the Liferay web framework. The tool is primarily designed for developing relatively small, linguistically-oriented vocabularies. In addition to testing whether various existing SKOS vocabularies could be loaded into the tool and rendered correctly, for each of the SKOS thesauri evaluated, we tested basic editing functionality (e.g. whether a new concept could be created and inserted into the existing thesauri, whether concepts could be deleted). The results of this evaluation are shown in Table 2. Note that even very large vocabularies (e.g. STW Thesaurus) could be edited successfully using the tool.

**Table 1:** General functioning evaluation

Thesaurus	Size	#Concepts	Loading Time	Hierarchy ^1^	PrefLabel ^2^	Save ^3^
STW Thesaurus	15MB	6584	40 sec	Y	Y	Y
NYT Subj Descriptions	1.4 MB	499	3 sec	Y	Y	Y
UK Archive Thesaurus	9.4 MB	13,976	120 sec	Y	Y	Y
Aus. Curr. Framework	312 KB	170	0.5 sec	Y	Y	Y
UNESCO Thesaurus	5.1 MB	44408	6 sec	Y	Y	Y

^1^Is the SKOS broader/narrower hierarchy rendered correctly? [Y or N]

^2^Are the prefLabels rendered correctly? [Y or N]

^3^Can the file be successfully edited, saved, then reopened? [Y or N]

**Table 2:** Editing functioning evaluation

Thesaurus	New Concept ^1^	Delete New Concept ^2^	Add/Edit PrefLabel ^3^	Add TopConcept ^4^
STW Thesaurus	Y	Y	Y	Y
NYT Subj Descriptions	Y	Y	Y	Y
UK Archive Thesaurus	Y	Y	Y	Y
Aus. Curr. Framework	Y	Y	Y	Y
UNESCO Thesaurus	Y	Y	Y	Y

^1^Can a new SKOS concept be created (Y/N)?

^2^Can a SKOS concept be deleted (Y/N)?

^3^Can a SKOS PrefLabel be added and edited (Y/N)?

^4^Can a SKOS Concept be added and edited (Y/N)?

### Qualitative evaluation

**Fig. 4:**
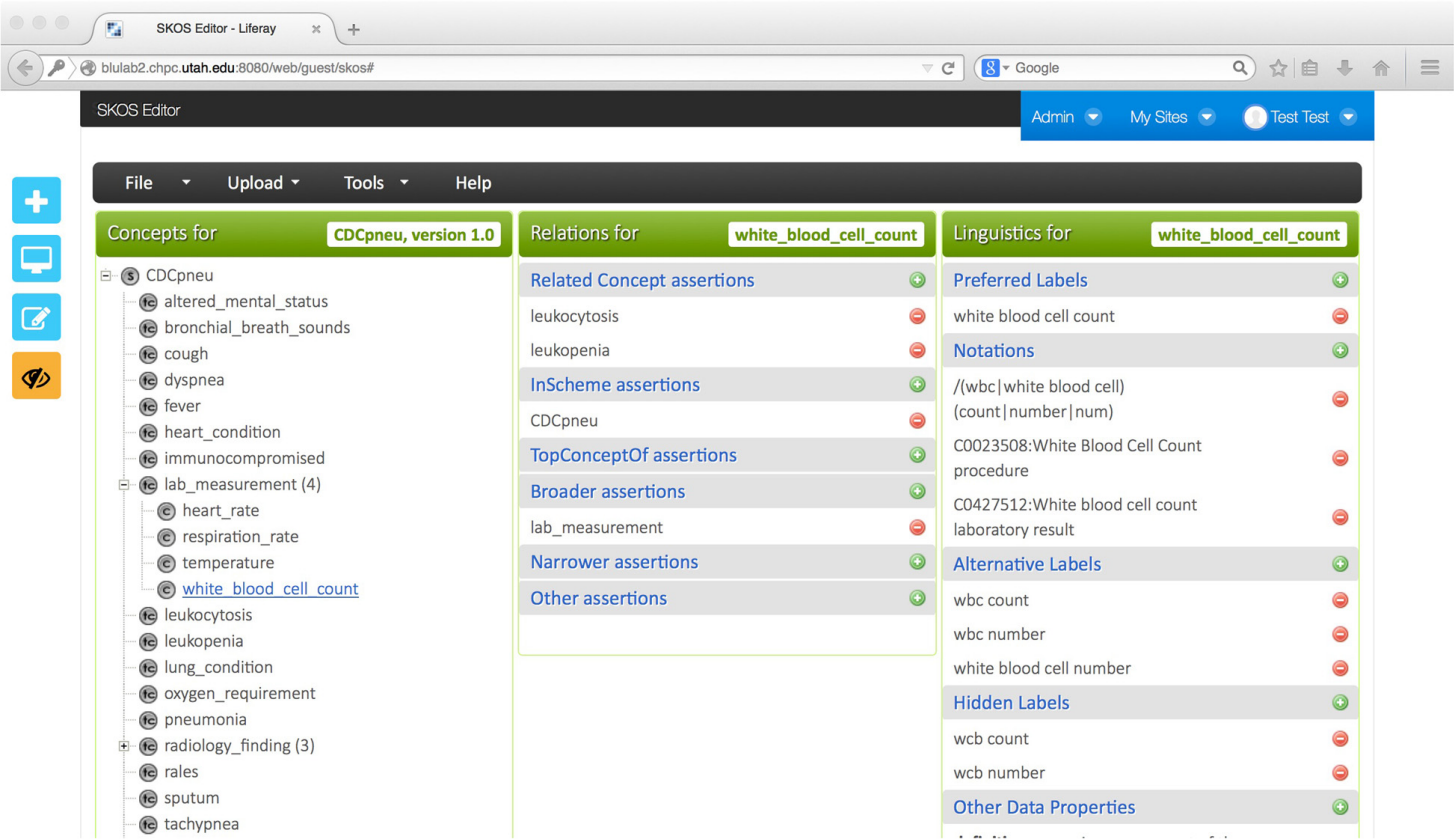
Pneumonia lexical resource based on Centers for Disease Control definition

**Fig. 5:**
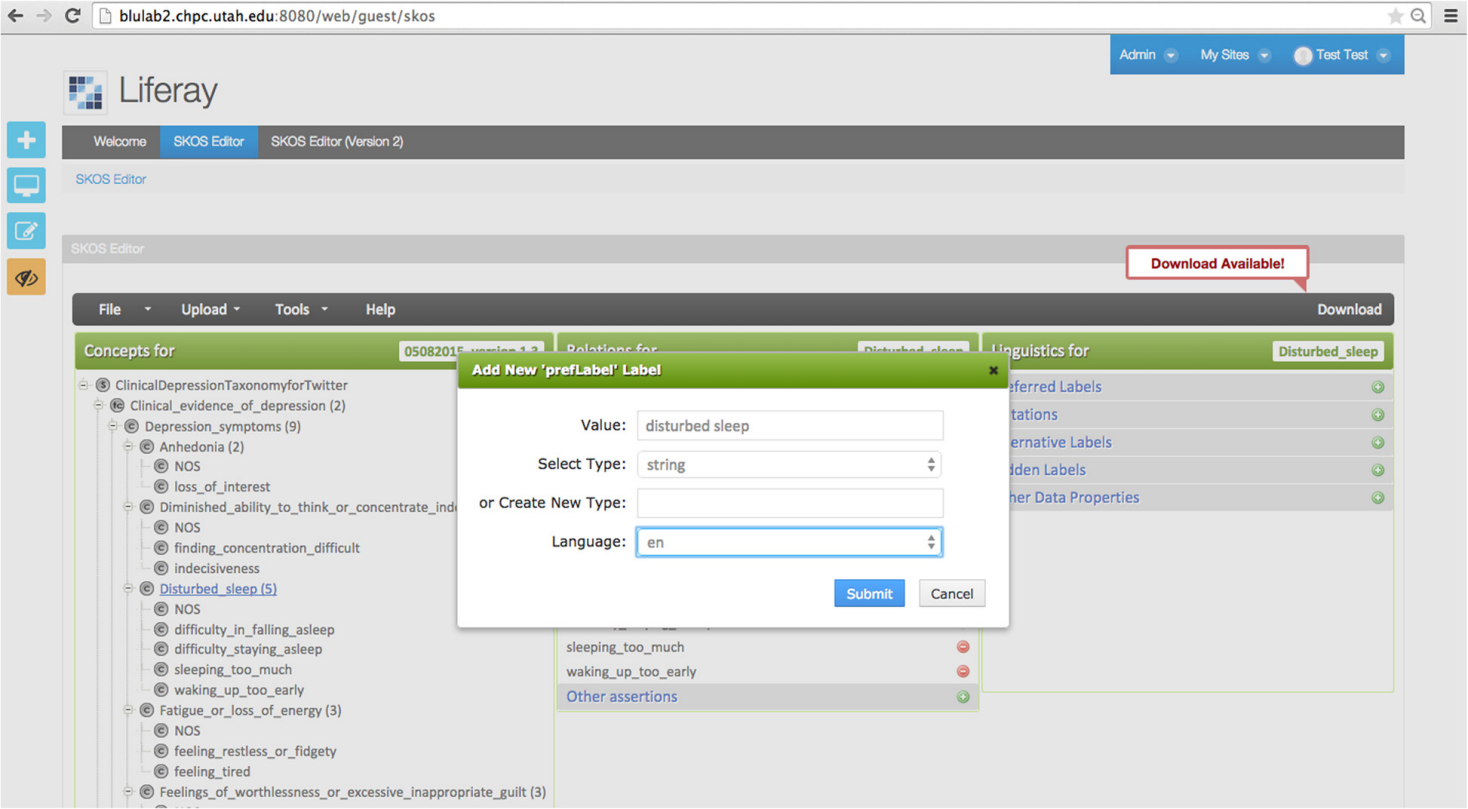
Building a depression lexicon – entering a preferred label

Our qualitative evaluation of the SKOS editor centred on two use cases. For the first use case, an experienced knowledge engineer (author Castine) used the SKOS editor to build a lexical resource to drive an EHR-oriented NLP algorithm based on the Centers for Disease Control pneumonia definition (see Fig. 4 for a screenshot of the resulting SKOS resource). The pneumonia resource took a total of 40 min to build using the Web SKOS Editor, as opposed to the Protégé SKOS Editor Plug-in, which took 45 min. Note that the knowledge engineer did not use the Wizard concept creation functionality, a tool which we believe is likely to expedite the concept hierarchy creation process substantially. For the second use case, an experienced NLP researcher (author Mowery) used the tool to develop a resource designed to map lexical cues to Diagnostic and Statistical Manual of Mental Disorders (DSM-5) diagnoses with the goal of facilitating the automatic identification of symptoms associated with depression from short, informal texts [21] (see Fig. 5 for a screenshot of the SKOS resource creation process). The depression resource took less than one hour to create, and it was reported that the Wizard greatly expedited the concept creation process. However, several enhancements were suggested, including the development of an auto-save feature, and the ability to configure default values for language labels (for example, default to English — @en — labels).

### Limitations

While the SKOS editor is suitable for building and curating special purpose SKOS vocabularies to run bespoke clinical NLP systems, it does have several limitations:

- It is not suitable for editing very large SKOS vocabularies
- As the tool is built around the SKOS API [9], some language features outside “core SKOS” [1] are not supported (e.g. skos:closeMatch, skos:relatedMatch).

### Future directions

Our long-term goal is to integrate the SKOS editor as a lexicon development and management module within a comprehensive platform for developing clinical NLP algorithms. As part of this long term goal — and informed by the comments and suggestions of our early users — we plan three major system enhancements:

- In the medium term, we plan to add multi-user functionality and collaborative editing to the system.
- We plan to include the ability to search other vocabularies – in particular the UMLS (Unified Medical Language System) [22] – from within the editor interface in order to expedite the synonym identification process.
- We plan to extend the current documentation and tutorial material

## Conclusions

The SKOS editor we have developed is – to the best of our knowledge – the first free, open-source, online, SKOS editor capable of creating, curating, versioning, and managing SKOS vocabularies. The editor is free to use^5^ and the source code is available under an Apache Version 2.0 License.

## Availability and requirements

**Project name:** Web-based SKOS editor**Project home page:** An instantiation of the tool is available at http://blulab2.chpc.utah.edu:8080/web/guest/skos. Source code is released under an open-source license and can be found at the University of Utah’s Biomedical Language Understanding Lab GitHub page https://github.com/Blulab-Utah**Operating system:** Multi-platform, browser-based**Programming languages:** Java, JavaScript**Other requirements:** No other requirements**License:** Apache 2.0 License**Any restrictions to use by non-academics:** No restrictions

## Endnotes

^1^ Note that additional SKOS tutorial material is available at: http://www.w3.org/2004/02/skos/references

^2^www.webcitation.org/6QmsQg41G

^3^www.webcitation.org/6Yfw7yX6b

^4^www.webcitation.org/6QmsXXNCc

^5^ SKOS editor URL: http://blulab2.chpc.utah.edu:8080/web/guest/skos

## Additional file

Additional file 1
**Tutorial.** (DOCX 2109 KB)

## Abbreviations

DSM-5: Diagnostic and Statistical Manual of Mental Disorders, 5th Edition
EHR: Electronic Health Record
NLP: Natural Language Processing
OWL: Web Ontology Language
RDF: Resource Description Framework
SKOS: Simple Knowledge Organization System
