# Supplementary material for: Developing a web-based SKOS editor
Source: J Biomed Semantics. 2016 Apr 4;7:5. doi: 10.1186/s13326-015-0043-z (PMC4819276; doi:10.1186/s13326-015-0043-z)
Supplement: Additional file 1 — Tutorial. (DOCX 2109 KB) [file 13326_2015_43_MOESM1_ESM.docx]

# Web SKOS Editor Tutorial

May 2015

Version 1.0

Mike Conway & Danielle Mowery

## Introduction

This brief tutorial is designed to help you get up and running with Web SKOS Editor, a tool designed to support the creation, development, curation, and versioning of small-to-medium sized lexicons using the SKOS (Simple Knowledge Organization System) W3C standard. We will work through some simple examples in order to help you become familiar with the interface. A minimal knowledge of SKOS is probably required to benefit from this tutorial (There are several tutorial introduction to SKOS available^[[1]](#footnote-1)^).

All examples used in this tutorial are derived from a use case that involves developing a taxonomy of lexical cues designed to support the automatic identification of symptoms associated with depression from short, informal texts.

## Setting up user account

The account set up process is straightforward. Please go to:

<http://blulab2.chpc.utah.edu:8080/web/guest/skos>

Make sure “SKOS Editor” and not “SKOS Editor (Version 2)” is highlighted. Version 2 is an experimental version of the system with untested features.

And click on the blue “Create Account” button in the middle of the screen


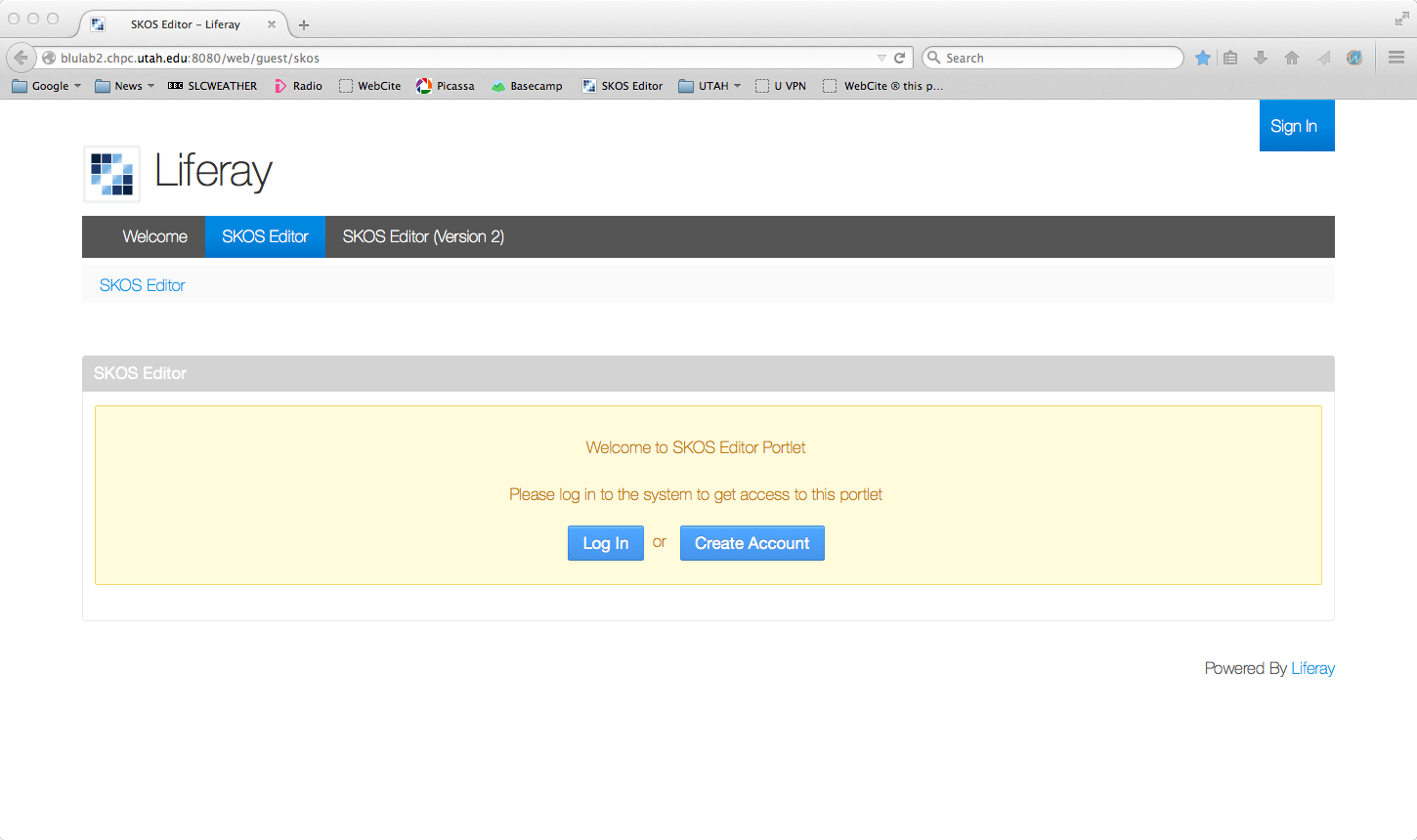


Then fill in your account details. Only the “First Name”, “Email Address”, and “Text Verification” are required.


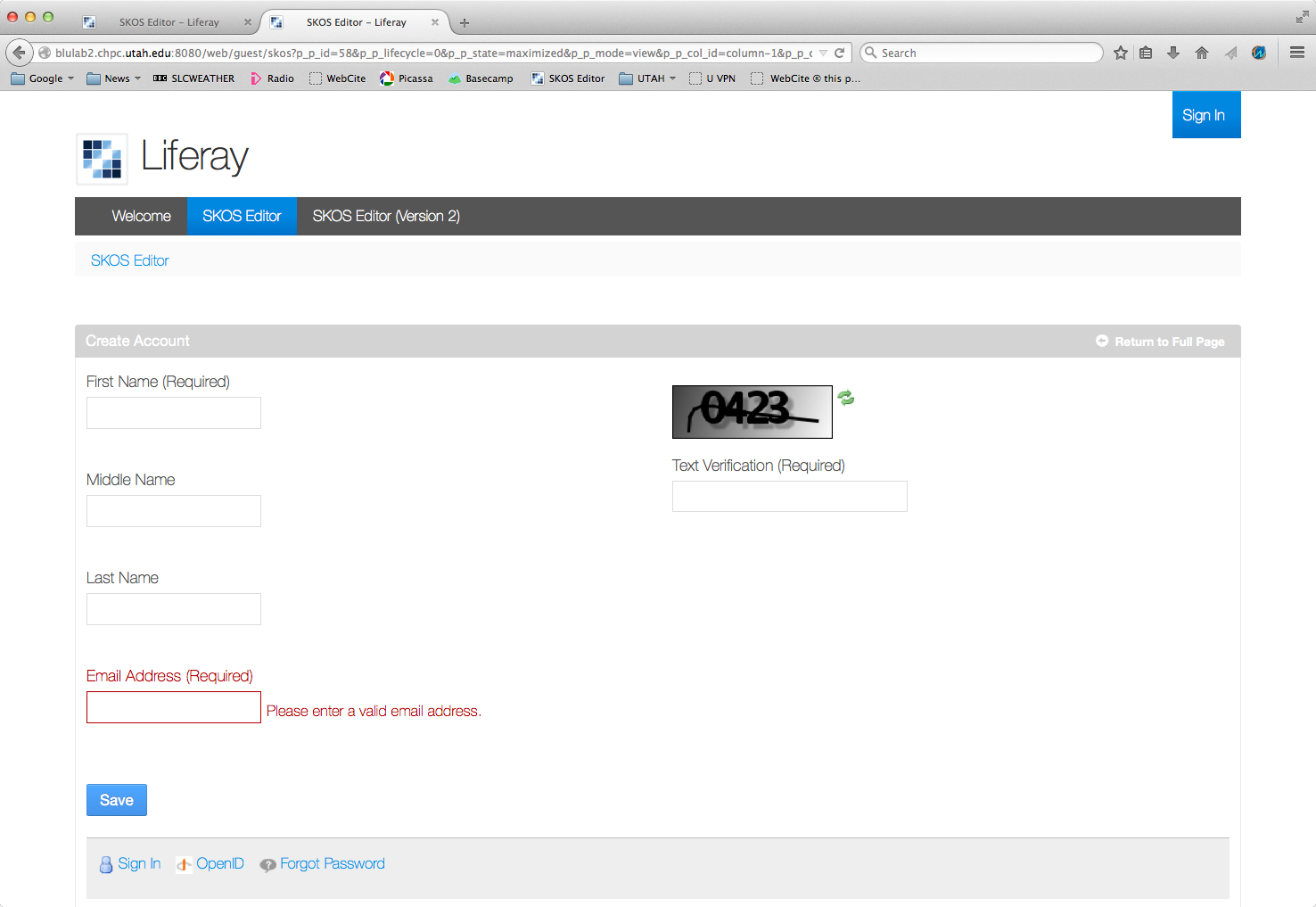


Then enter the email address and password supplied to log in.


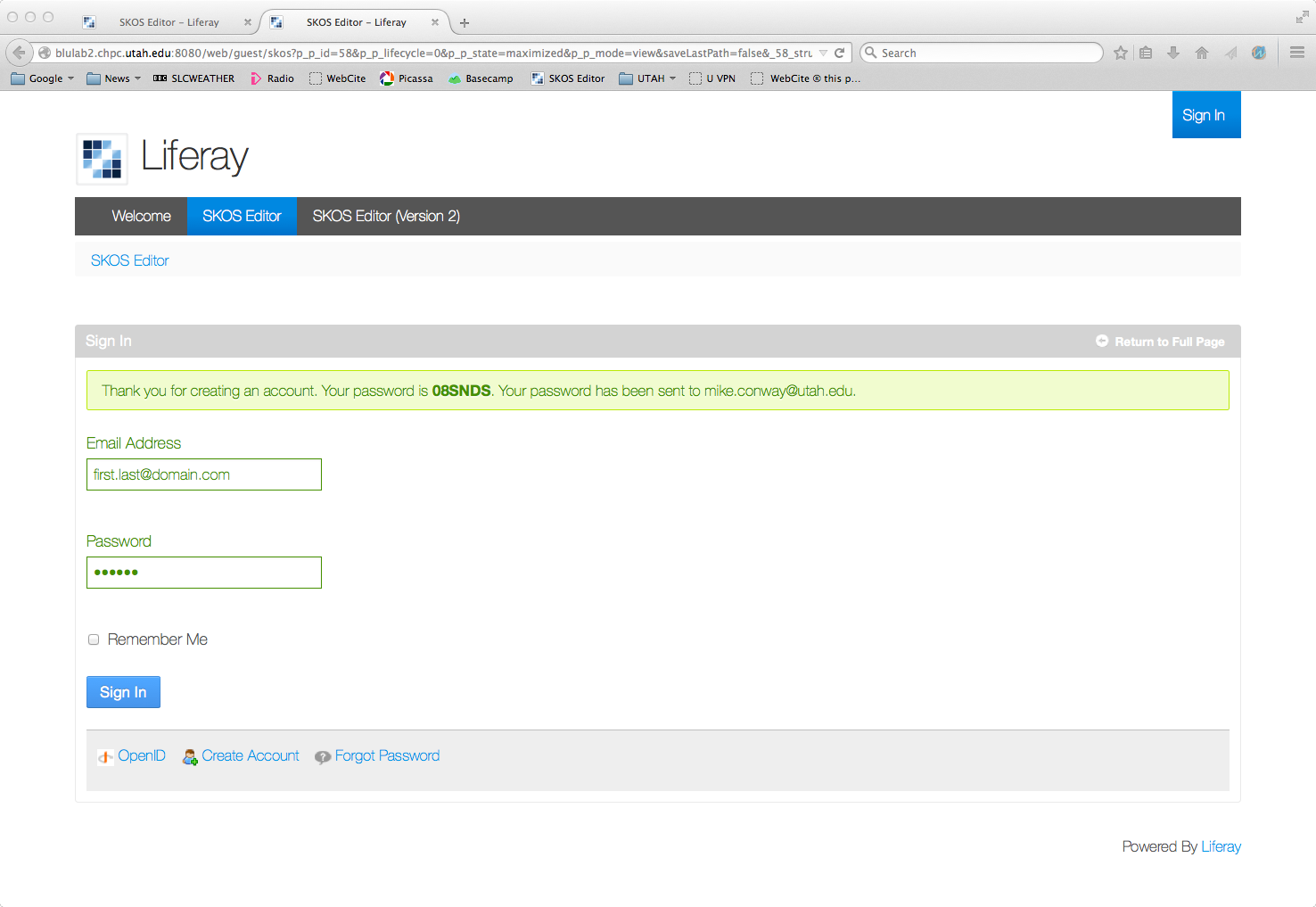


## Creating a new SKOS thesaurus

To create a new SKOS thesaurus, go to “File” ⇒ “Create New” (see figure below).


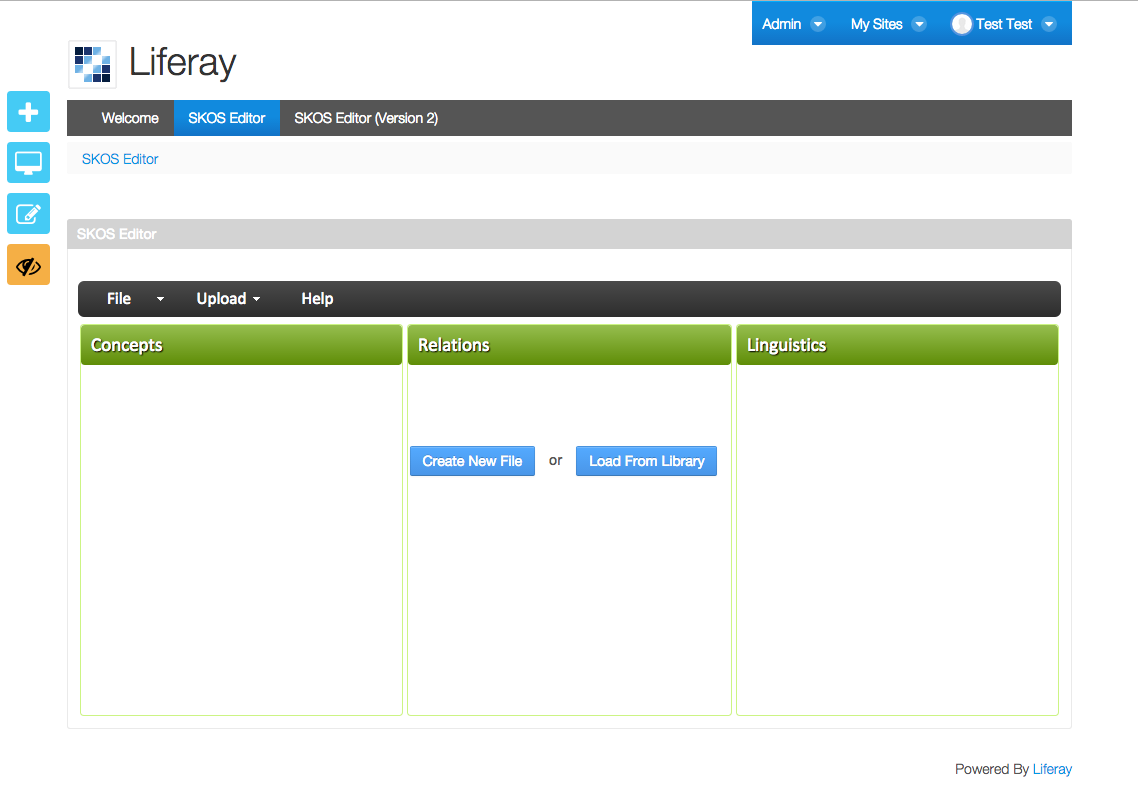


Then you will be presented with a “Create New SKOS Knowledge Base”. The figure below shows how to name and create metadata for the SKOS thesaurus.


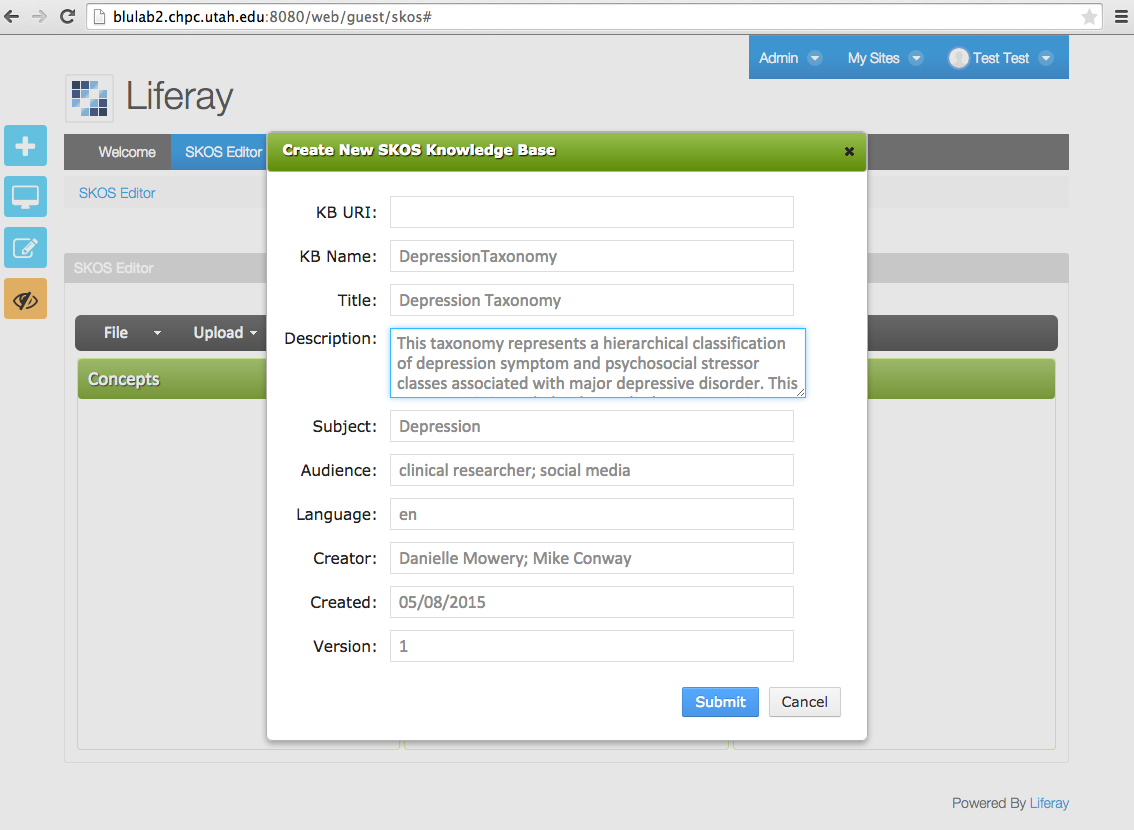


After pressing “Submit”, a new SKOS thesaurus is generated (see Figure below).


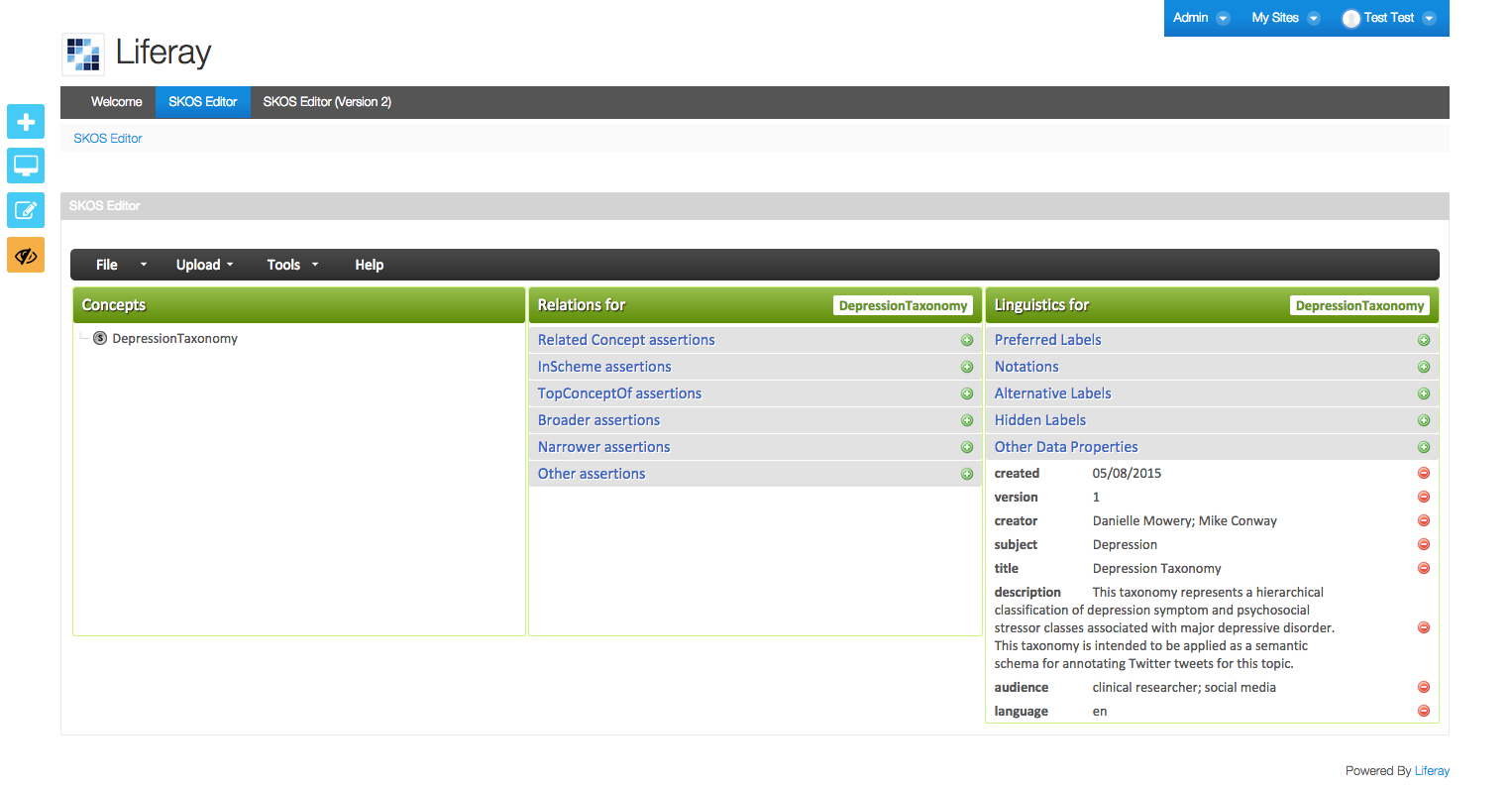


Note that if you do not wish to create a new SKOS file from scratch, an example file can be loaded by selecting “Upload” ⇒ “From URL”, then selecting option “i” from the dialog box.

## Populating the thesaurus using the “Create Class Hierarchy” Wizard

Concepts can be entered using a GUI-based “Create Class Hierarchy” Wizard to support the rapid creation of SKOS concept hierarchies. The Wizard can be accessed via “Tools” ⇒ “Create Class Hierarchy” (see below)


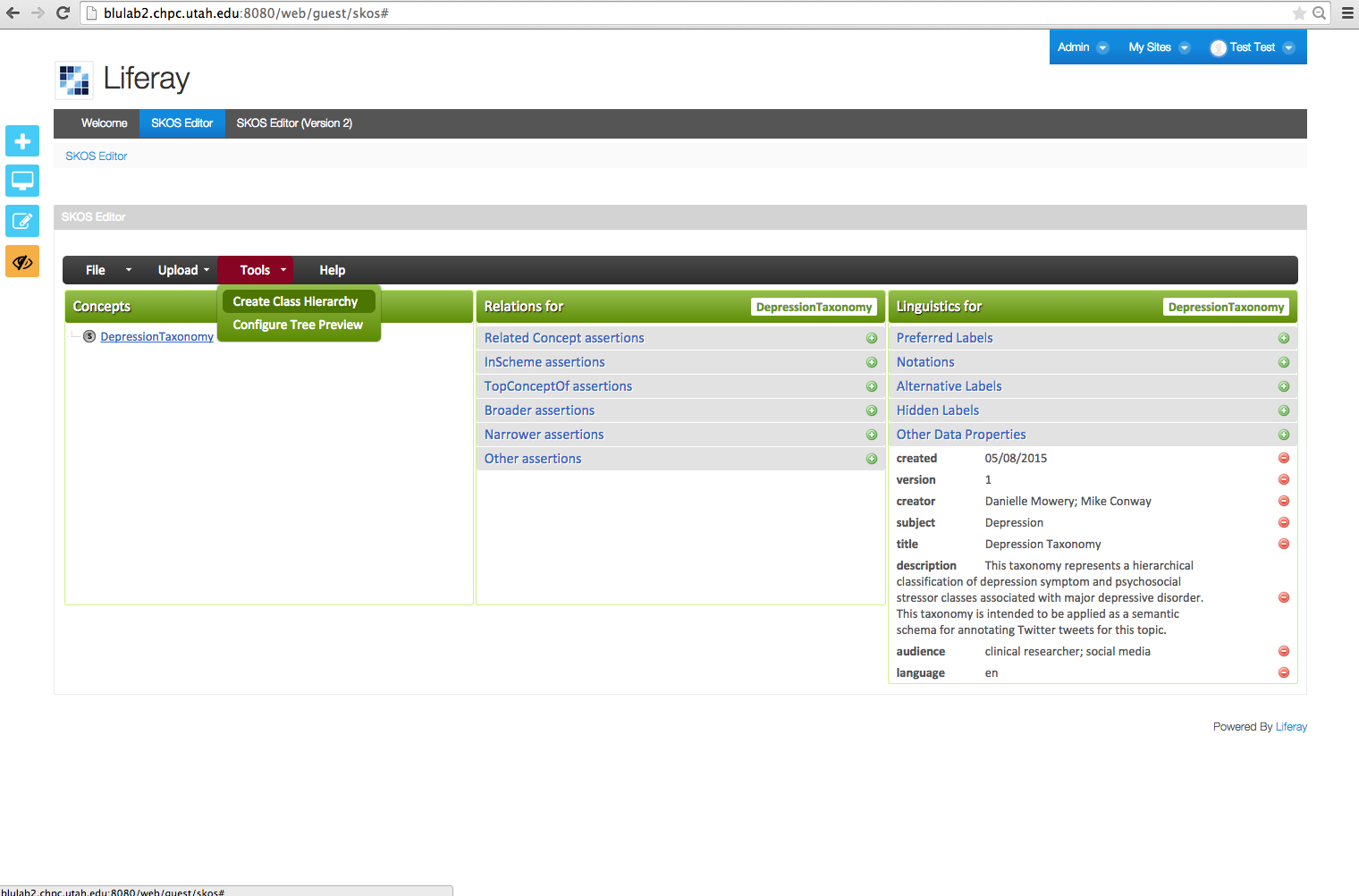


The desired concept hierarchy can be entered using a tab-indented text format (see “Hierarchy” text box below)

##
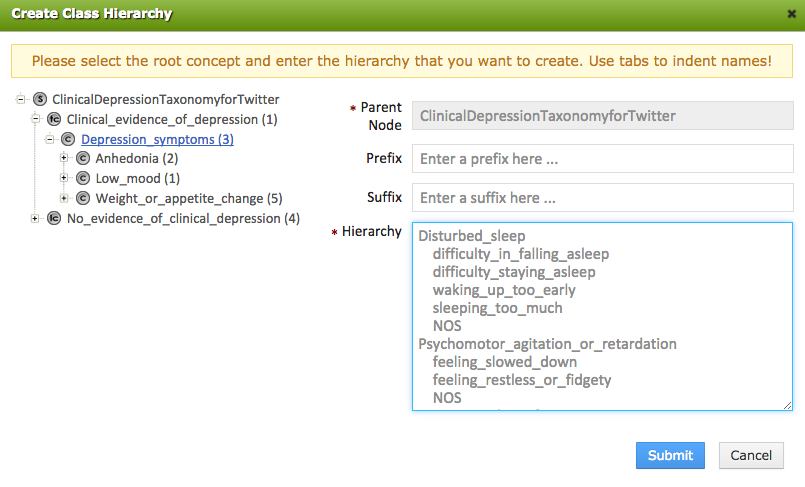


## Editing preferred labels

Once a concept hierarchy has been developed, it is straightforward to add preferred, alternative, and hidden labels for each concept. SKOS preferred labels can be added by clicking on the + character next to “Preferred Labels” in the Linguistics Panel (rightmost panel). The figure below shows an example preferred label creation dialog box for the “Disturbed_sleep” concept. Note that the language (e.g. “en”, “fr”, “es”, “jp”) can be selected from a drop-down list.


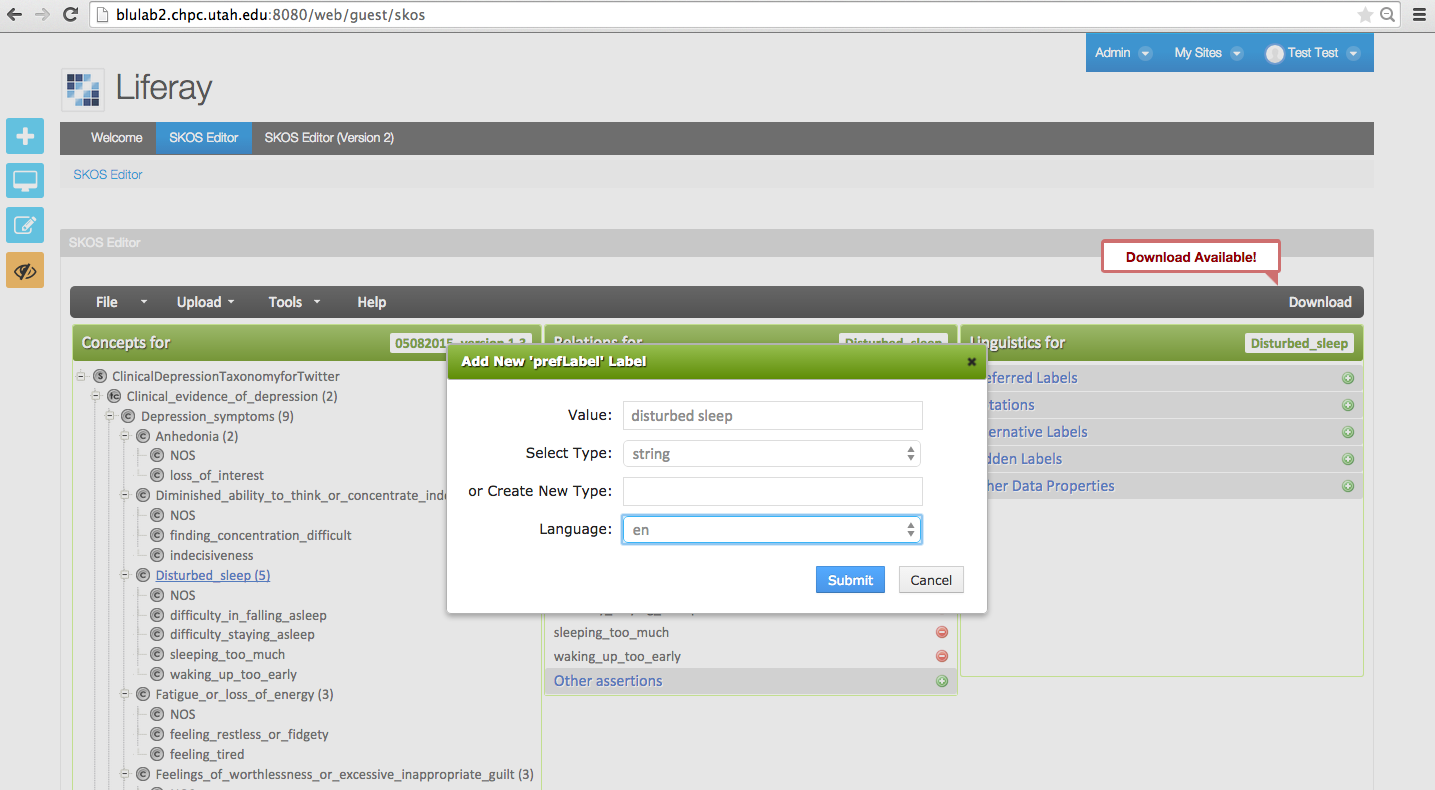


## Downloading the edited SKOS thesaurus

The edited thesaurus can be downloaded using the “Download” button (top right corner of the interface – see below).


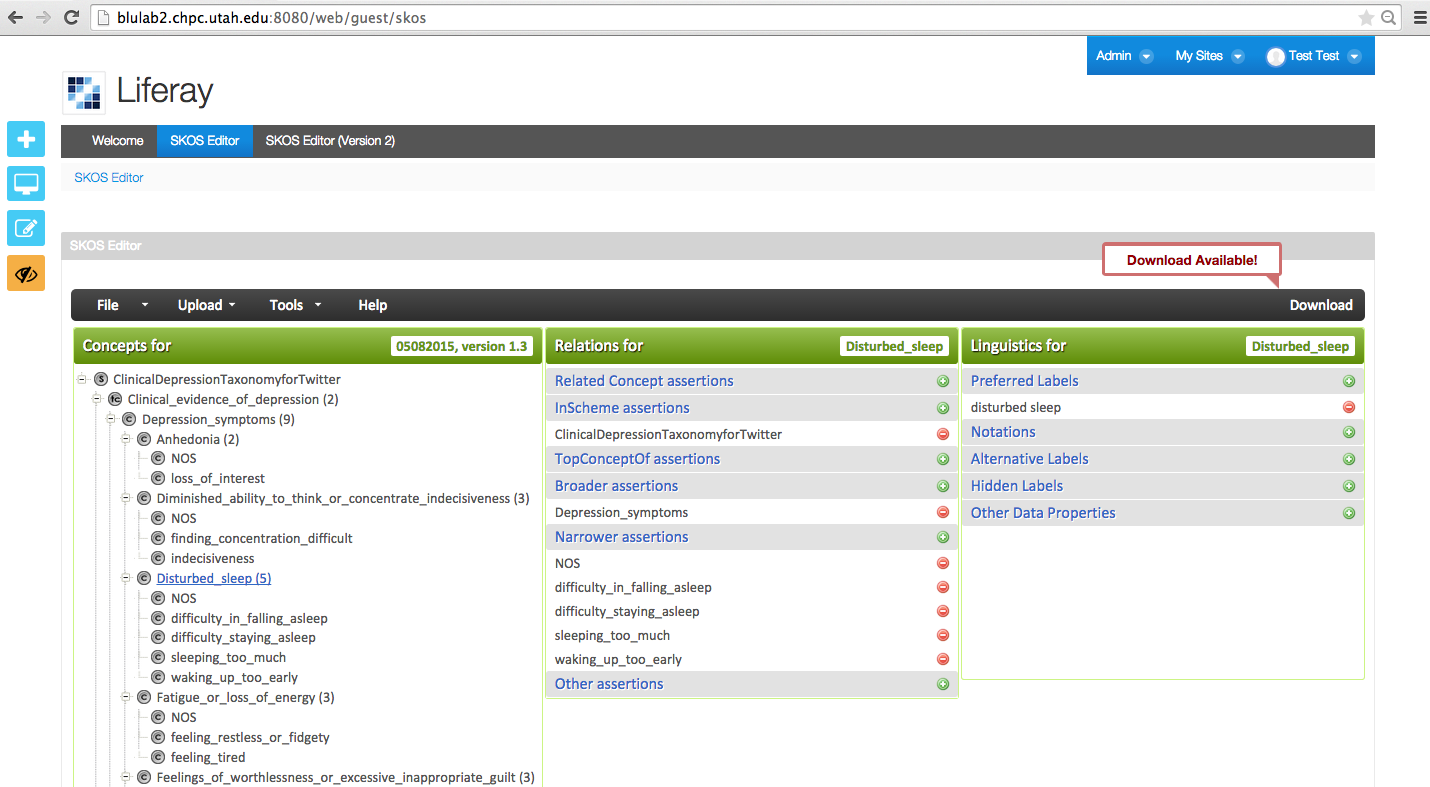


The downloaded OWL/XML is generated using the SKOS and OWL APIs (see screenshot below).


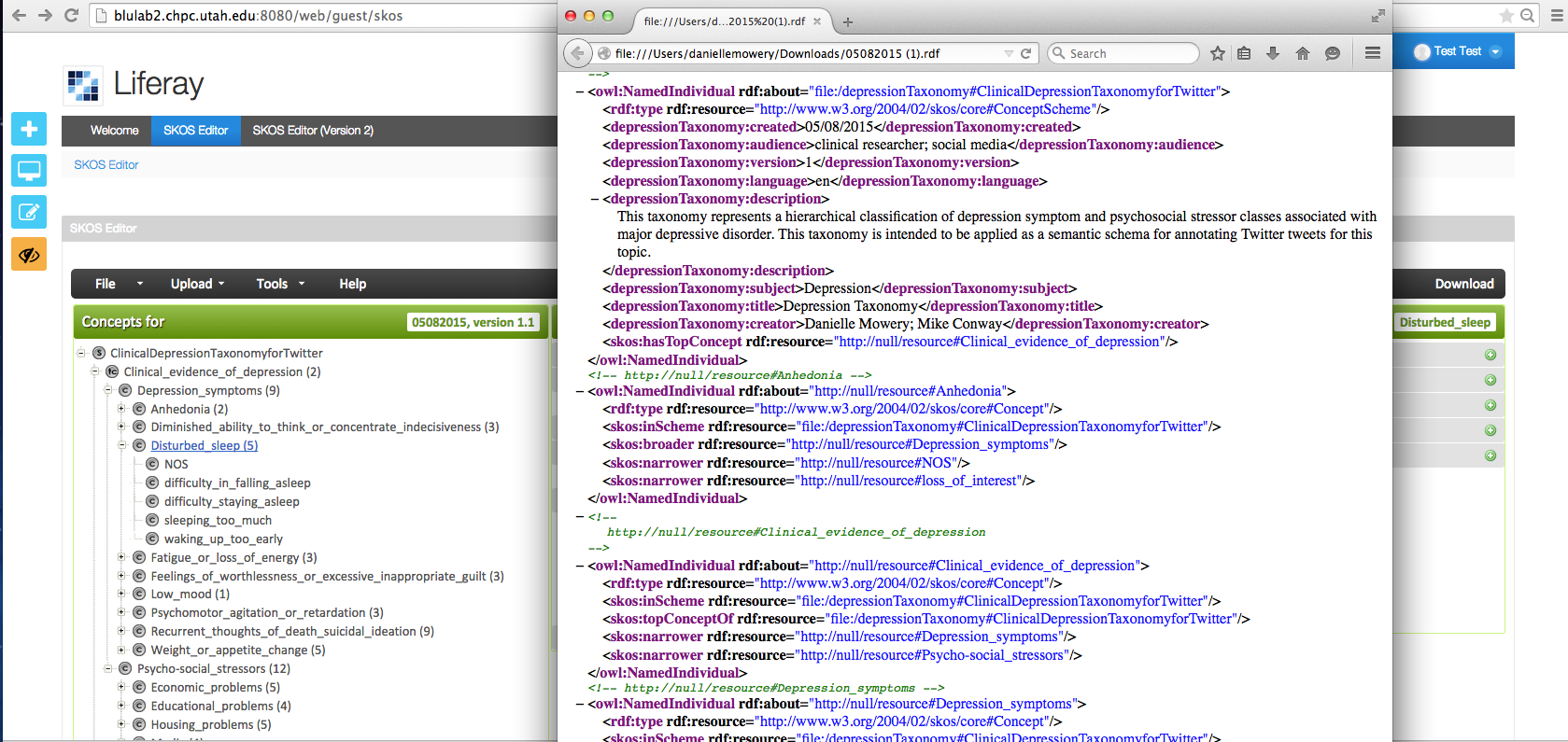


1. SKOS tutorial: http://www.w3.org/2004/02/skos/references [↑](#footnote-ref-1)
